# A Clinical Study to Evaluate Sensorineural Hearing Loss in Diabetic Patients Presenting to a Tertiary Healthcare Center

**DOI:** 10.7759/cureus.80664

**Published:** 2025-03-16

**Authors:** Fathima Jabeen, Nithya Narayanan

**Affiliations:** 1 ENT and Head and Neck Surgery, Apollo Hospitals, Chennai, IND; 2 Otolaryngology - Head and Neck Surgery, Apollo Hospitals, Chennai, IND

**Keywords:** diabetes mellitus, high-frequency hearing loss, microangiopathy, sensorineural hearing loss, stria vascularis

## Abstract

Background

Diabetes mellitus is a chronic disease that causes an array of dysfunctions, mainly because of its neuropathic and microvascular complications. This study was based on the hypothesis that these complications can lead to hearing loss in diabetic patients. The study also evaluates other influences on hearing loss in diabetic patients.

Objectives

The objective is to assess the prevalence of sensorineural hearing loss among diabetic patients and to analyze whether other factors contribute to worsening hearing loss among diabetics.

Methods

A prospective study was conducted among diabetic patients aged 35-60. A total of 86 patients were involved in this study.

Results

The prevalence of hearing loss among diabetic people in our study was 70.9%. Most hearing loss patients had mild sensorineural hearing loss, with a significant high-frequency slope or dip. There is a higher chance of hearing loss among diabetic patients if they have associated hypertension as well. No association between HbA1c level and duration of diabetes was established. As the age of the diabetic patient rises, there is a notable increase in the chance of having hearing loss as well, even though it was not statistically proven.

Conclusions

The hyperglycemic effects causing cochlear dysfunction start early in the phase of diabetes, and many people are unaware of their hearing deprivation. This might be because most of the affected people have mild sensorineural hearing loss. Early detection and intervention are needed to prevent the progression of this morbid condition.

## Introduction

The three parts of the ear are the external ear, the middle ear, and the inner ear. The middle ear begins from the tympanic membrane, which contains the three little bones: the malleus, incus, and stapes. They move sound waves from your tympanic membrane to your inner ear. Damage to the cochlea, or auditory nerve, causes sensorineural hearing loss (SNHL). There can be multiple etiological factors that cause inner ear injury leading to SNHL, like noise, ototoxic drugs, age-related degeneration, interference with vascular supply, aberrant metabolic activity, head injury, as well as genetic abnormalities that alter the inner ear's anatomical architecture [1]. Sound is transmitted to the inner ear as vibration of the cochlear fluids and the hair cells. Mechanical stimulation of the hair cells converts these vibrations into electrical impulses. These impulses are subsequently transmitted from the cochlea through the vestibulocochlear nerve (CN VIII) to the auditory cortex [2]. The highly vascularized stria vascularis tissue, which borders the cochlea's lateral wall, secretes the endolymph for the scala media and preserves the ionic balance around the outer and inner hair cells [3]. The cochlea receives its blood supply from two tiny terminal arteries. The tiny diameter of the arterial supply channels and the absence of collateral blood supply make the cochlea vulnerable to damage from a range of vascular insults [4-6].

Diabetes and hearing impairment are two extremely common incapacitating illnesses. Due to its high metabolic activity, the auditory pathway is vulnerable to hyperglycemia. Numerous processes, such as microangiopathy, neuropathy, and oxidative stress-induced damage, have been proposed to explain the onset of hearing loss in diabetic patients [7]. Research on the potential connection between diabetes and hearing loss is frequently skewed by exposure to noise, the use of ototoxic drugs, and age. The intricate connection between diabetes and hearing loss is examined in this study so that early detection is possible. We also attempted to study the pathophysiology of the disease process to know where and when to intervene and halt the disability progression so that every diabetic could get a better quality of life.

## Materials and methods

A prospective study was conducted in the ENT Outpatient Department in a tertiary care hospital in Chennai, India, from June 2023 to June 2024 on diabetic patients between 35 and 60 years old who came for health checkups. Debilitating patients, patients with a history of noise exposure, ear trauma, ototoxic drug usage, ear discharge, hearing loss before diabetes diagnosis, hearing loss from childhood, patients with Meniere’s disease, conductive hearing loss, tympanic membrane perforation, and newly detected diabetic patients were excluded from the study. A total of 86 subjects who fulfilled the selection criteria were included in the study and evaluated. Ethical approval was sought from the institutional review board before initiating the study (IEC-BMR Application Number: AMH-DNB-053/06-23).

Sample size 

Since the primary objective of the study was to determine the prevalence of hearing loss in diabetic patients, the sample size was calculated by assuming the expected proportion as 66% (diabetic patients with hearing loss), 10% precision, and a 95% confidence interval, based on the study conducted by Srinivas et al. [8].

Methodology

Written and informed consent was obtained from each subject. They were assessed based on demographic details and detailed history, including diabetic history, medical history, personal history, and drug history, and underwent a general examination and examination of both ears. Based on these details, people who met the exclusion criteria were excluded from the study. The people who were not excluded, with or without hearing loss, were further evaluated by a pure tone audiogram. The grading system for audiometry followed in our institution is: normal hearing: 0-25 dB, mild hearing loss: 26-40 dB, moderate hearing loss: 41-55 dB, moderately severe hearing loss: 56-70 dB, severe hearing loss: 71-90 dB, and profound hearing loss: >90 dB.

Statistical analysis

Mean (SD) and frequency (percentage) were used to describe the summary data. The continuous factors were compared by using the Student’s t-test/Mann-Whitney U test. Chi-square/Fisher’s exact test was used to find out the association between categorical factors. P-values < 0.05 were considered statistically significant. All the statistical analysis was done using SPSS software 28.0 (IBM Corp., Armonk, NY).

## Results

The total number of people enrolled in the study was 86 (n = 86). We categorized the study population into three age groups: 35-40 years, 41-50 years, and 51-60 years. Most of the diabetic patients belonged to the older age group, i.e., 51-60. Maximum incidence of hearing loss was seen among the age group of 51 to 60 years (P-value = 0.149). There were 64 males and 22 females in the study. No significant association between gender and hearing loss was noted.

People were again categorized into three groups based on the duration of diabetes: ≤5 years with 42 (48.8%) patients, 6-10 years with 30 (34.9%) patients, and >10 years with 14 (16.3%) patients. There is significant hearing loss in all three groups, and the maximum incidence of hearing loss was higher among the patients with diabetes duration between six and 10 years, which was 80%, followed by 66.7% for the age groups less than or equal to five years and the least incidence of 64% in more than 10 years of diabetes. However, the P-value was> 0.05 and thus was not statistically significant. Out of the 86 diabetic patients, 43 (50%) patients had diabetes mellitus only with no other comorbidity, while the remaining 43 (50%) patients had hypertension as a comorbidity along with diabetes. Among both the diabetic and hypertensive subjects, 81.4% had hearing loss when compared with 60.5% of diabetes-only subjects (P-value = 0.056). In our study, 31 patients (36%) had HbA1c values less than 7, and 29 patients (33.7%) had HbA1c values 7-8, and 26 patients (30.2%) had HbA1c > 8. The incidence of hearing loss was higher in patients with HbA1c values between 7 and 8, which is 24 patients; for patients with HbA1c values < 7, it was 20; and for patients with HbA1c values > 8, the number of patients with hearing loss was 17. P-value > 0.05 indicates statistically not significant (Table 1).

**Table 1:** Association of age, gender, duration of DM, other comorbidities and HbA1c values with hearing loss. PTA: pure tone audiometry, DM: diabetes mellitus, HTN: hypertension

Parameters	PTA (n (%))		Total	P-value*
	Hearing Loss,	Normal Hearing,	(n=86)	
	(n=61)	(n=25)		
Age (in years)				
35 – 40	6 (50.0)	6 (50.0)	12	
41 – 50	9 (64.3)	5 (35.7)	14	0.149
51 – 60	46 (76.7)	14 (23.3)	60	
Gender				
Males	45 (70.3)	19 (29.7)	64	>0.99
Females	16 (72.7)	6 (27.3)	22	
Duration of DM				
(in years)				
≤5	28 (66.7)	14 (33.3)	42	
6 – 10	24 (80.0)	6 (20.0)	30	0.393
>10	9 (64.3)	5 (35.7)	14	
Comorbidities				
DM alone	26 (60.5)	17 (39.5)	43	0.056
DM + HTN	35 (81.4)	8 (18.6)	43	
HbA1C				
<7	20 (64.5)	11 (35.5)	31	
7 – 8	24 (82.8)	5 (17.2)	29	0.226
>8	17 (65.4)	9 (34.6)	26	

Out of the 86 diabetic patients, 33 (38.4%) had hard of hearing, and 53 (61.6%) did not have any hearing complaints. All the patients enrolled in this study underwent pure tone audiometry, irrespective of the presence or absence of symptomatic hearing loss. Out of the 86 patients evaluated, it was observed that 61 (70.9%) patients had hearing loss, and 25 (29.1%) patients had normal hearing.

Among the 61 patients with hearing loss, the incidence of mild SNHL was found to be the highest, which constituted 25 patients (41%), followed by 16 patients (26%) with normal hearing and dip at high frequencies. This is followed by seven patients (11.4%) with moderate SNHL. Severe SNHL and profound hearing loss were reported in six patients each (10%), and the least incidence was for moderately severe SNHL, i.e., only one patient (Table 2).

**Table 2:** Distribution of severity of hearing loss. SNHL: sensorineural hearing loss

Pure tone audiometry	No of patients (n=61)	Percentage
Mild SNHL (0-25 dB)	25	41
Moderate SNHL (26-40 dB)	7	11.40
Moderately severe SNHL (41-55 dB)	1	1.60
Severe SNHL (56-70 dB)	6	10
Profound HL (71-90 dB)	6	10
Normal hearing with dip at high frequency	16	26

Out of the 25 mild SNHL cases, 18 patients (72%) had a high-frequency dip/slope, and seven patients (28%) had hearing loss at all frequencies. All the patients with moderate, moderately severe, severe, and profound hearing loss had all the frequencies affected. Patients with normal hearing with a slope at high frequency were 16 (Table 3).

**Table 3:** Association between frequency affected and severity of hearing loss. PTA: pure tone audiometry, SNHL: sensorineural hearing loss

	PTA (n=61)						
Frequency affected	Mild SNHL, n(%)	Moderate SNHL, n(%)	Moderately severe SNHL, n(%)	Severe SNHL, n(%)	Profound HL, n(%)	Normal hearing with loss at high frequency, n(%)	P-value
High-frequency dip/sloping pattern	18(72)	0	0	0	0	16(100)	<0.001
Hearing loss in all frequencies	7(28)	7(100)	1(100)	6(100)	6(100)	0	
Total	25	7	1	6	6	16	

## Discussion

According to our study, the overall prevalence of hearing loss was 70.9%. When compared with other studies, prevalence varies from 55% to 74% [8-11]. Our study did not draw any firm conclusions about the influence of the gender of diabetic patients on hearing loss.

Many published literature reports an increase in hearing loss among older diabetics [8,12-14]. We have taken the upper age limit of the study population as 60 years to prevent presbycusis (age-related hearing loss) as a confounder in our study. Despite this fact, there was more hearing loss among the 51-60 age group. The older diabetic group can have an enhanced effect of age-related oxidative stress and microvascular changes in the inner ear structures.

Unlike other published studies, we were not able to prove the link between the increased risk of hearing loss and the increased diabetes duration [8,10]. This might be because the least number of patients belonged to the > 10 years diabetic duration group (lengthiest diabetic duration) in the study.

This study did not demonstrate any association between HbA1c levels and hearing loss, which is in contrast with the study by Srinivas et al. [8]. Even though diabetes is controlled, moderately controlled, or poorly controlled, the pathophysiological changes of diabetes affect the inner ear, its microvasculature, sensory cells, and supporting cells.

In our study, we observed an increased incidence of hearing loss in diabetic patients with hypertension when compared with diabetic patients without hypertension (P-value = 0.056). About 81.4% of diabetic and hypertensive subjects had hearing loss when compared with 60.5% of diabetes-only subjects. Hypertension is an established risk factor for circulatory failure. Hypertension thickens the basement membrane of the stria vascularis, which is connected to hypoxic conditions and also causes artery constriction and a downstream reduction in blood flow. Major consequences of the combined effect of diabetes mellitus and hypertension include microvascular insults that disrupt oxygen exchange, ion transport, and blood flow and hyperglycemia, leading to neural degeneration affecting the nerve conduction pathway. This result is supported by Shafiepour et al. and Bener et al. [7,14]. However, Samelli et al. did not find any association between hypertension and hearing loss [15].

In our study, the maximum number of hearing loss cases was mild SNHL, which constituted 41% of the total hearing loss. 72% of mild SNHL had a dip/slope at high frequency. These findings are supported by many previous studies by Edgar et al., Ren et al., and Akinpelu et al. [12,13,16]. We classified the hearing loss people into two categories: those with a high-frequency slope/dip and those with all frequencies affected. It was found that 55.7% had dip/slope at high-frequency patterns, and 44.3% had all frequencies affected. All the hearing loss patients in the high-frequency pattern belonged to the mild SNHL and normal hearing with dip/slope in the high-frequency group. Several other investigations had similar findings, showing that the most damaged hearing function was high-frequency hearing [10].

In our study, more than 60% of the diabetic patients who came for the health check did not have any hearing complaints when asked. Irrespective of the hearing complaints, everyone underwent pure tone audiometry. After doing the hearing evaluation, we found that out of the 61 subjects who showed a hearing loss in the audiogram, only 33 patients actually complained of any hearing loss, whereas the remaining 28 patients, i.e., 46%, did not have any hearing complaints.

The inner ear vessels are affected by a microangiopathic process, and the auditory nerve is affected by neuropathy that results in SNHL. The pathological changes start even in the early stages of diabetes, which is shown by the fact that, even at the minimum diabetic duration (less than or equal to five years), there were a notable number of hearing loss cases. Patients with longer diabetic duration had a significant proportion of hearing loss, but the total number of people with longer diabetic duration was less in our study. This explains the reason why the association with diabetic duration was not statistically significant.

In diabetic patients, the initial findings may be, firstly, a mild SNHL and, secondly, normal hearing with a dip at high frequencies that may be unnoticeable by the patients. This points to the importance of early identification of the condition and prompt treatment. Interventions that aim to control and reverse the microvascular and neuropathic changes are to be tried. Based on these facts, we have put forward a few recommendations: Using pure tone audiometry to screen all newly diagnosed diabetic patients; perform annual pure tone audiometry for diabetic patients regularly, even if the patient does not report a hearing deficit so that we can provide early treatment; use of a high-frequency audiometer for assessing diabetic patients, because high frequencies tend to get affected first in the course of the disease.

This study has potential limitations. First, a larger study population would have increased the study's accuracy. Second, since we did not include a control group - that is, individuals without diabetes - a comparison is not feasible. Additionally, noise exposure assessments were based on the participant’s recall. It is therefore subject to a recall bias. Despite these limitations, the study's strengths lie in its rigorous methodology and randomization, significantly contributing to the literature. Addressing these limitations in future research will allow for a more comprehensive understanding of the relationship between hearing loss and diabetes.

## Conclusions

The overall prevalence of hearing loss was 70.9%. Most of them had mild SNHL. There were more cases of hearing loss with a high-frequency dip. From our study, we could hypothesize that diabetes mellitus is a risk factor for hearing loss. We deduced that cochlear dysfunction is a significant consequence of diabetes mellitus based on the results of the current investigation.
